# Association of Gestational Age in a Full Range With Childhood Overweight and Obesity: A Systematic Scoping Review

**DOI:** 10.1111/obr.13939

**Published:** 2025-05-10

**Authors:** Yuantao Su, Marini Ahmad Suhaimi, Manish Prasad Gupta, Wenchong Du, Jing Hua

**Affiliations:** ^1^ Department of Women's and Children's Health Care, Shanghai Key Laboratory of Maternal Fetal Medicine, Shanghai Institute of Maternal‐Fetal Medicine and Gynecologic Oncology, Shanghai First Maternity and Infant Hospital, School of Medicine Tongji University Shanghai China; ^2^ NTU Psychology, School of Social Sciences Nottingham Trent University UK

**Keywords:** childhood obesity, early‐term, gestational age at birth, overweight, post‐term, preterm, scoping review

## Abstract

**Background:**

Existing evidence shows inconsistent associations between preterm birth and childhood obesity. The impact of full gestational age on the risk of childhood obesity and overweight remains unclear.

**Objectives:**

This systematic scoping review aims to comprehensively explore the relationship between gestational age at birth across the entire spectrum and childhood overweight and obesity.

**Method:**

A thorough search of online databases (PubMed, Ovid Medline, and Google Scholar) covering the period from January 2000 to September 2024 was conducted using selected keyword strings following the PRISMA‐SCR guidelines. Studies investigating childhood overweight or obesity as either a primary or secondary outcome in association with various degrees of births were included.

**Result:**

Twenty‐eight studies met the inclusion criteria. Substantial evidence linked preterm births to childhood overweight/obesity. However, the available evidence for term‐born and post‐term‐born children was limited, showing mixed results regarding their relationship with childhood overweight and obesity.

**Conclusion:**

The available evidence regarding the association between different gestational age categories and childhood overweight/obesity is limited. This review underscores the importance of implementing primary prevention strategies during early childhood and highlights the need for further research to enhance our understanding of the nuanced relationship between gestational age and childhood overweight/obesity.

**Abbreviations**
BMI: body mass index
EAR: early adiposity rebound
FFM: free fat mass
FMI: fat mass index
FT: full term
GA: gestational age
GDM: gestational diabetes mellitus
HDL: high density lipoprotein
LDL: low density lipoprotein
LP: late preterm
MD: mean difference
MP: moderately preterm
OWO: overweight/obesity
OR: odds ratio
PRISMA‐ScR: Preferred Reporting Items for Systematic Reviews and Meta‐Analyses Extension for Scoping Reviews
REE: resting energy expenditure
REE: resting energy expenditure
RR: relative risk
SDS: standard deviation scores
SFT: skin fold thickness
VCAM‐1: vascular cell adhesion molecule‐1
VP: very preterm
WHO: World Health Organization
WHR: waist–hip ratio
WHtR: waist–height ratio

## 1 Introduction

Overweight and obesity in children represent significant global public health challenges [1] that can often persist into adulthood [2, 3, 4]. According to the World Health Organization (WHO), overweight and obesity are characterized by the abnormal or excessive fat accumulation, and a body mass index (BMI) exceeding 25 is classified as overweight, while a BMI exceeding 30 is classified as obese. These childhood conditions is associated with a higher likelihood of long‐term adult‐related mortality and morbidity [5], as well as increased risks of developing chronic diseases, such as Type II diabetes, cancer, and cardiovascular disease [6, 7, 8, 9]. Although the prevalence of childhood overweight and obesity is more pronounced in developed countries, it is also rising in developing nations, indicating a global trend that requires urgent attention [10, 11].

Research has shown that children with obesity are twice as likely to become adults with obesity compared to children without obesity [12]. The intrauterine and early postnatal periods are critical for the development of adult obesity [13, 14], with recent epidemiological studies focusing on early‐life events, including prenatal and fetal periods [15]. The likelihood of developing obesity, particularly in children of parents with obesity, can often be predicted from birth or even pre‐pregnancy [16, 17]. Several studies have indicated that neonatal adiposity may serve as a marker of adequate intrauterine growth and, consequently, a potential risk factor for future obesity [18, 19, 20, 21, 22].

Pregnancy represents a crucial period in human development. Deviations from normal gestational age, including early‐term, preterm, and post‐term births, have been associated with acute and long‐term adverse health outcomes in children [23, 24, 25, 26, 27, 28, 29, 30, 31, 32, 33, 34, 35, 36, 37, 38, 39, 40]. Growth patterns in preterm infants differ from those of full‐term children throughout childhood and school years [25, 26, 27, 28, 29, 30, 31]. The relationship between preterm birth and childhood obesity has been extensively studied, yielding mixed results; some report a positive association [32, 33, 34, 35, 36], while others find a negative correlation [37, 38, 39, 40]. Further research has examined the effects of gestational age, extending beyond preterm to post‐term births, and also report inconsistent results [41, 42, 43]. This variability underscores the inconclusive nature of the current literature on the relationship between the full range of gestational ages and childhood overweight and obesity, emphasizing the need for additional research.

The aim of this scoping review is therefore to systematically evaluate the existing literature on the association between the full spectrum of gestational age and the incidence of childhood overweight and obesity. Specifically, we wanted to determine whether children born at different degrees of prematurity (< 37 weeks) and post‐term (≥ 42 weeks) are at a higher risk for childhood overweight and obesity compared to children born at term (37–41 weeks). This investigation is crucial for addressing the discrepancies observed in previous studies and aims to enhance our understanding of how gestational age impacts the risk of childhood obesity, which is crucial for developing targeted interventions and policies to address this global health issue.

## 2 Method

The scoping review followed the Preferred Reporting Items for Systematic Reviews and Meta‐Analyses Extension for Scoping Reviews (PRISMA‐ScR) Checklist [44]. The methodological framework developed by Arksey and O'Malley, which involves a six‐stage process, was utilized for this scoping review [45]. The objective of this review was to address the research question: What is the evidence regarding the effects of gestational age across the complete range on childhood overweight/obesity (OWO).

The identification of relevant studies and the recognition of the limited number of published systematic and scoping reviews addressing the research questions were the focus of this phase. To ensure a comprehensive and broad search, we selected three databases for our systematic searches: PubMed, Ovid Medline, and Google Scholar. PubMed and Ovid Medline were chosen for its reputation as a comprehensive source for biomedical and life sciences literature, ensuring a high level of scientific rigor and relevance. Google Scholar was included to complement them. This dual approach allows for the capture of a more extensive and diverse body of literature may offer additional insights and perspectives not covered in traditional databases.

Systematic searches were conducted using these databases, focusing on relevant studies published in English between January 1, 2000, and September 30, 2024. The inclusion of studies from 2000 onwards was chosen to align with the period when significant advancements in obesity research and perinatal care were documented, providing a contemporary context for our review. The search terms employed included “obesity”, “fatness”, “overweight”, “heavyweight”, “longitudinal growth”, “BMI”, “adiposity”, “fat mass”, “free fat mass”, “metabolic risk”, combined with terms such as “perinatal risk factor”, “gestational age”, “gestation at birth”, “preterm child”, “term child”, and “post‐term child”. The search was conducted exclusively in English, reflecting the international scope of the research and the desire to capture a global perspective on the topic (See Appendix S1 for details). To enhance the methodological clarity of this scoping review, the characteristics of the included studies are detailed, covering study designs, sample sizes, geographical locations, and specific demographic groups targeted. This expansion provides a comprehensive understanding of the studies' contexts and the applicability of their findings. Specifically, the review delineates the age groups examined (i.e., whether children or adolescents) and the diversity of the settings.

The title, abstract, and full paper of the articles were then screened. Inclusion and exclusion criteria were predetermined, and further screening was conducted accordingly. The inclusion criteria consisted of: (1) studies examining childhood obesity or similar terminologies (e.g., childhood overweight, childhood adiposity, fatness, heavy weight, longitudinal growth of the body, high BMI); (2) studies investigating whether gestational age at birth is a risk factor for obesity. The review categorized gestational age as preterm (< 37 weeks), full‐term (including early‐term [37–38 weeks], completely full‐term [39–40 weeks], and late‐term [41 weeks]), and post‐term (≥ 42 weeks). The current scoping study encompassed both experimental and non‐experimental primary studies, while systematic reviews and meta‐analyses were also considered; however, none of these studies met the inclusion criteria; (3) the participants' age was between 3 months and 18 years old. In studies where a single data point within this age range met the requirements, it was also included for analysis.

The following criteria were applied to exclude studies: (1) studies that did not investigate the association between gestational ages and childhood obesity as an outcome; (2) narrative and systematic reviews; and (3) studies focused solely on associations other than gestational ages, such as maternal obesity, maternal gestational diabetes mellitus (GDM), birth weight, small or large gestational age, breastfeeding practices, and nutritional supplementation during infancy. Non‐English articles with an English abstract were also excluded from this review.

The title and abstract of all articles identified through the search strategy underwent screening to determine their eligibility for inclusion. Authors YS conducted the initial screening process. In cases of uncertainty, records were reviewed by MA, and a consensus was reached through mutual agreement on the inclusion of full‐text articles. Any discrepancies were resolved by WD and JH to ensure a final consensus on the included articles.

Data extraction for this review followed a systematic approach that considered the gestational age of the child at birth (preterm, full‐term, post‐term), the association analyzed (which category of gestational age at birth was examined in relation to childhood overweight and obesity), the outcomes (how childhood overweight or obesity is associated with each category of gestational age at birth), and the study design. The following data were systematically extracted: gestational age of the child population at birth, the specific category of gestational age (preterm, full‐term, post‐term), whether a significant association with childhood obesity was found, study design, and the level of evidence. The level of evidence was appraised using the Oxford Levels of Evidence, as defined by the Centre for Evidence‐Based Medicine [46]. This system categorizes evidence into four recommendation levels (A, B, C, D) and five reliability levels (I, II, III, IV, V), allowing for a structured assessment of evidence based on its methodological quality and relevance to different research domains such as prevention, diagnosis, prognosis, treatment, and harm. This structured evaluation aids in synthesizing research findings more reliably and applicably across studies in the review.

**FIGURE 1:**
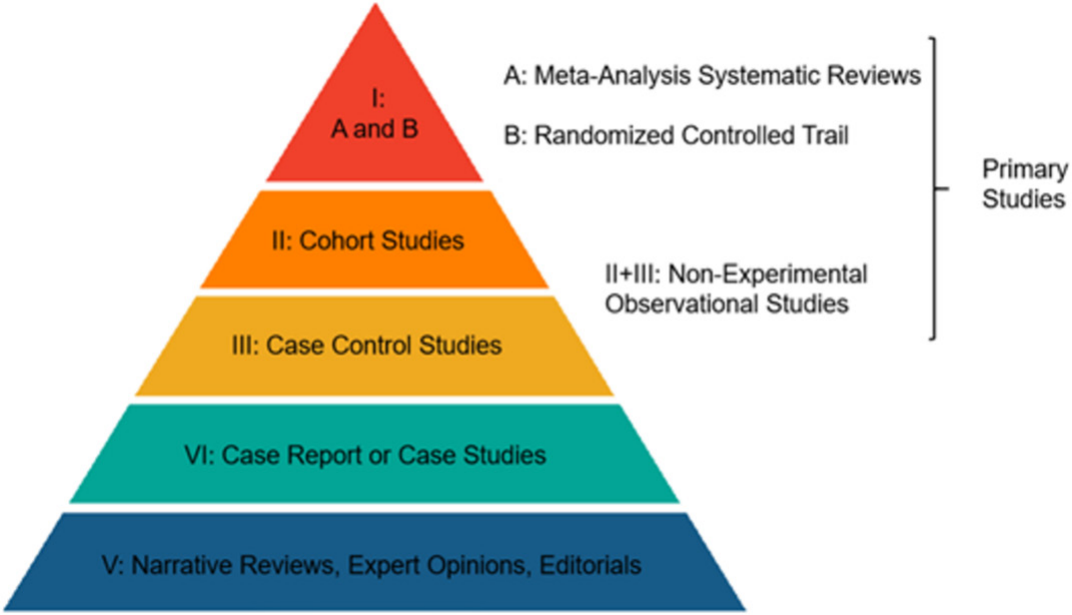
Study types and levels of clinical evidence.

Data sorting, summarization, and reporting were performed based on the primary outcomes of full gestational age on adiposity and childhood obesity. The review highlighted findings from studies examining the risk of childhood obesity in preterm, term, and full‐term infants. The discussion and summary of the results were based on the characteristics of the study population, outcome measures, and level of evidence (Figure 1).

## 3 Results

### 3.1 Description of Included Studies

**FIGURE 2:**
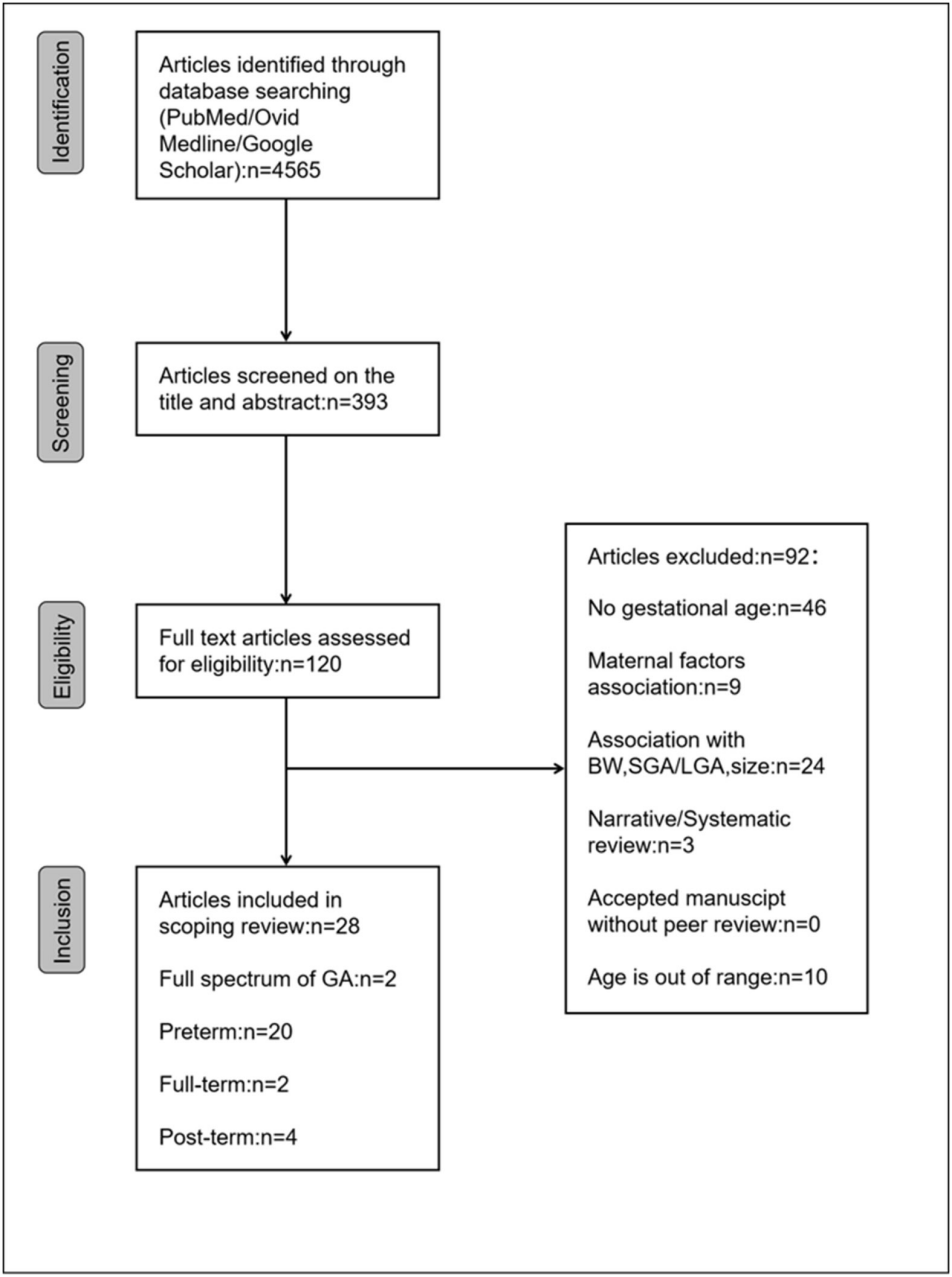
Flow chart of the search strategy.

A total of 28 articles met the inclusion criteria for this scoping review. Specifically, 20 articles examined the association between gestational age and childhood obesity in preterm birth, while 2 articles focused on full‐term gestational age. Additionally, 4 articles investigated the relationship between gestational age and childhood obesity in post‐term birth, and 2 articles reported on the full spectrum of gestational age (Figure 2). In terms of geographical distributions of the population, among the 28 included studies, 4 were conducted in the Italy and China, and 3 each in the USA and Sweden. Furthermore, 2 studies each were carried out in United Kingdom, Israel, and Poland, while 1 study each originated from Australia, Germany, Spain, France, India, New Zealand, and Brazil. There's another one that covers multiple regions.

Regarding the study design, 24 studies were cohort studies, indicating an evidence level of 2. The remaining 4 studies were observational studies, with an evidence level of 3 (Table 1).

**TABLE 1:** Study characteristics of the included studies with their evidence of association.

Author	Country	Study design	Level	Sample size	Age	Covariates	GA at birth	Definition of overweight/obesity	Association
Roswall J et al 2012 [26]	Sweden	Cross sectional cohort study	2	152 moderately preterm	1.5, 2, 3, 4.5,5 years	Birth weight SDS, birth length SDS, gender	Moderately preterm infants (32–37 weeks' gestation) vs. reference population ( > 33 weeks' gestation)	BMI (overweight: >25 kg/m^2^)	Negative (defined as overweight: preterm = 4.9% vs. reference = 13.3%, *p* < 0.05; student's t‐test: preterm BMI SDS at 2 years = 0.45 ± 1.2 SDS, *p* < 0.001; preterm BMI SDS at 5 years = 0.49 ± 1.2 SDS, *p* < 0.001)
Bortolotto CC et al 2021 [27]	Brazil	Cohort study	2	36, 167 preterm and 1361 term	6 years	Birth weight, family income, height, maternal education, maternal age, maternal smoking during pregnancy, pregestational BMI, skin color	Preterm infants (≤ 33 weeks' gestation, 34–36 weeks' gestation) vs. term infants (37–41 weeks' gestation)	BMI *z*‐score	Negative (BMI (kg/m^2^) for boys: at ≤33 GA β = −0.78(−1.36, 0.20), at 34–36 GA β = −0.69(−0.99, −0.40), *p* < 0.001) Irrelevant (BMI (kg/m^2^) at girls: at ≤33 GA β = −0.31(−1.03,0.42), at 34–36 GA β = −0.28(−0.56, 0.00), *p* = 0.116)
Perenc L et al 2019 [37]	Poland	Observational study	3	61 preterm	5–8 years	Apgar score at fifth minute of life, birth weight, number of fetuses, number of pregnancies, sex	Preterm infants (between 22 and 37 weeks' gestation) vs. the reference system (the Fenton preterm growth chart)	BMI *z*‐score	Negative (BMI (kg/m^2^): Z = −5.38, *p* < 0.001)
Giannì ML et al 2008 [38]	Italy	Prospective cohort study	2	45 preterm and 40 term	4.8–6.6 years	Prenatal, postnatal variables	Preterm infants (< 34 weeks' gestation and ≤ 1800 g) vs. term infants (with birth weight between 10th and 90th on north Italian growth charts)	BMI	Negative (BMI (kg/m^2^) at school age: preterm = 15.5 ± 1.9, term = 16.8 ± 2.9, *p* < 0.05)
Giannì ML et al 2015 [39]	Italy	Longitudinal, observational study	3	63 preterm and 61 term	5 years	Basic characteristics, maternal and paternal BMI	Preterm infants (< 32 weeks' gestation with a birth weight of <1500 g) vs. term infants (37–41 weeks' gestation with birth weight between the 10th and 90th percentile on World Health Organization growth chart)	BMI	Negative (BMI of boys (kg/m^2^): preterm = 15.1 ± 1.0, term = 15.9 ± 1.2, *p* < 0.05)
Huke V et al 2013 [40]	Germany	Prospective cohort study	2	116 preterm and 120 term	5–7 years	Age, family history of cardiovascular disease, SGA, sex, maternal and paternal BMI, diet	Preterm infants (24–33 weeks' gestation) vs. term infants (37–41 weeks' gestation)	BMI percentile	Negative (percentile value for BMI: preterm = 36 ± 29, term = 49 ± 28, *p* < 0.001, MD = ‐12.95 (−20.36, ‐5.54))
Beltrand J et al. 2012 [41]	Sweden	Prospective cohort study	2	37 post‐term and 488 term	16 years	Actual age at measurement, auxologic data, birth weight, parental BMI	Post‐term infants (≥ 42 weeks' gestation) vs. term infants (37–41 weeks' gestation)	BMI percentile (overweight: > 85th; obesity: > 95th)	Positive (BMI: +3.7 kg/m^2^ in post‐term boys (*p* < 0.01); Obesity: 29% (post‐term boys) vs. 7% (term boys) (*p* < 0.01); Overweight/obesity: 47% (post‐term boys) vs. 13% (term boys) (*p* < 0.01))
Suhaimi MA et al. 2024 [42]	China	Cohort study	2	7314 post‐term and 133,688 term	3–6 years	Child characteristics (breast feeding longer than 6 months), family characteristics(mother's and father's BMI)	Post‐term infants (≥ 42 weeks' gestation) vs. term infants (37–41 weeks' gestation)	BMI *z*‐score (for 3–4 years: overweight: 2SD < to < 3SD; obesity: ≥ 3SD; for 5–6 years: overweight: 1SD <to < 2SD; obesity: ≥ 2SD; for 3–6 years: thinness: < −2SD)	Positive (obesity: postterm RR = 1.46(1.29,1.65 ), overweight/obesity: postterm RR = 1.27(1.18,1.36 ), thinness: postterm RR = 1.13(1.06,1.20))
Ayyavoo A et al. 2013 [43]	New Zealand	Observational study	3	36 post‐term and 41 term	4–11 years	Age, birth weight SDS, birth order, ethnic composition, sex ratio, mean parental BMI, maternal pre‐ pregnancy BMI	Post‐term infants (≥ 42 weeks' gestation) vs. term infants (38–40 weeks' gestation)	BMI, body fat, FFM, truncal fat	Positive (BMI SDS: postterm = 0.30, Term = 0.11, *p* = 0.45, Body fat: postterm = 22.9%, term = 19.9%, *p* = 0.014, FFM: postterm = 77.1%, term = 80.1%, *p* = 0.014, Truncal fat: postterm = 21.2%, term = 18.0%, *p* = 0.017)
Giannì ML et al 2012 [47]	Italy	Cohort study	2	49 late preterm and 40 term	3 months	Child basic subject characteristics, maternal factor (BMI), nutritional practices	Late premature infants (34–36 weeks' gestation) vs. term infants (37–41 weeks' gestation)	Weight, length, head circumference, mean fat mass values	Irrelevant (weight: late preterm = 6050 ± 485, term = 5978 ± 580, length: late preterm = 59.6 ± 2.1, term = 60.6 ± 2.5, head circumference: late preterm = 40.4 ± 1.2, term = 39.9 ± 1.3, fat mass values: late preterm = 26.7 ± 4.3, term = 27.1 ± 3.9)
Fewtrell MS et al 2004 [48]	UK	Cohort study	2	497 preterm and 95 term	8–12 years	Age, level of physical activity, pubertal status, sex, neonatal diet	Preterm infants (< 37 weeks' gestation and with birth weight < 1850 g) vs. term infants	BMI, FMI	Negative (BMI (kg/m^2^): preterm = 17.5 ± 2.8, term = 18.2 ± 3.0, *p* < 0.05, slaughter equations: FMI (kg/m^2^): preterm = 3.34 ± 1.62, term = 3.93 ± 2.01, *p* < 0.005)
Regnault N et al. 2010 [49]	France	Prospective cohort study	2	1418 term	3 months	Gestational weight gain, heights of the mother and father, maternal age at delivery, method of feeding at 1 and 3 months, maternal tobacco use, parity, maternal pre‐pregnancy BMI, paternal BMI, sex	Term (≥ 37 weeks' gestation)	Weight‐growth velocity, length‐growth velocity	Negative (gestational age for each additional week: weight‐growth velocity at 3 months = −0.6 ± 0.1, *p* < 0.0001; length‐growth velocity at 3 months = −0.01 ± 0.002, *p* < 0.0001)
Scheurer JM et al 2017 [50]	USA	Cohort study	2	20 preterm and 51 term	3–4 months, 4 years‐preschool age	Age at visit 3, birth weight, maternal education, race, sex	Premature infants (< 35 weeks' gestation between 10th and 90th percentile on Fenton preterm growth curve at birth) vs. full‐term born infants (between 10th and 90th percentile on World Health Organization growth curve)	BMI, body fat	Irrelevant (body fat(%) at 3–4 months: preterm = 27.1 ± 2.17, term = 24.0 ± 1.03, *p* = 0.29; BMI (kg/m^2^) at 4 years‐preschool age: preterm = 15.1 ± 1.38, term = 15.6 ± 1.08, *p* = 0.13)
Baldassarre ME et al 2020 [51]	Italy	Prospective population based longitudinal study	2	100 preterm	3, 6, 9, 12, 15, 18, 24 months, and 3,4,5,6,7 years	Birth weight classification, duration of breastfeeding, parental education, parental BMI, sex, type of delivery, type of feeding at 6 months of age, timing of introduction of solid food	Preterm infants (23–36 weeks' gestation)	EAR	Irrelevant (GA:EAR = 34 ± 2, NO EAR = 34 ± 2, *t* = 0.19, *p* = 0.85)
Hui LL et al 2015 [52]	China	Cohort study	2	295 late preterm and 6872 term	1, 14 years	Highest parents' education attainment, mother's place of birth, pregnancy characteristics, presence of birth defects, sex, infant feeding, parent information	Late premature infants (34–36 weeks' gestation) vs. term infants (37–41 weeks' gestation)	BMI *z*‐score, mean weight, WHR, WHtR	Negative (mean weight at 12 months (Referenced to standard): late preterm = −0.16, term = 0.06, *p* < 0.001) positive (14 years, late preterm: BMI *z*‐score β = 0.13(−0.02,0.27), WHR *z*‐score β = 0.16(0.02,0.29), WHtR *z*‐score β = 0.25(0.11,0.38))
Jabakhanji SB et al 2018 [53]	Ireland	Cohort study	2	10,377 infants	9 months, 3 years, 5 years	Availability of green and play spaces, birth‐related, caregiver weight status, ethnicity, household structure, infant feeding, perceived neighborhood safety, race and region, socioeconomic, sex, sleeping hours	Very early preterm(<33 weeks' gestation), early preterm (33–36 weeks' gestation), term (37–41 weeks' gestation), postterm (≥ 42 weeks' gestation)	BMI percentile	Negative in preterm (BMI change at 9 months: very early vs. term β = −2.00 (−2.3, −1.69); early vs. term β = −1.09 (−1.23, ‐0.94)) positive in postterm (BMI change at 9 months: postterm vs. term β = 0.14(0.04, 0.24))
Tang J et al. 2022 [54]	China	Cohort study	2	2369 post‐term	4–7 years	Calendar year of birth, maternal and paternal sociodemographic characteristics, maternal lifestyle, maternal clinical parameters (BMI at enrollment), offspring characteristics	Post‐term infants (42–46 weeks' gestation) vs. term infants (37–41 weeks' gestation)	BMI *z*‐score (overweight/obesity: > + 1SD; thinness: <‐1SD)	Negative (mean ± SD BMI‐for‐age z score:−0.16 ± 1.06 mean difference: −0.11(−0.15, −0.06); overweight/obesity: postterm RR = 0.82(0.72,0.94), thinness: postterm RR = 1.18(1.09,1.28))
Spiegler J et al. 2020 [55]	UK	Cohort study	2	18,818 children	3–14 years	Child factors, diet, maternal obesity, parental education, sport habits	Very preterm (24–31 weeks' gestation), moderately preterm (32–33 weeks' gestation), late preterm (34–36 weeks' gestation) vs. term infants (≥ 37 weeks' gestation)	BMI	Negative (VP vs. FT at age 3, *p* < 0.001, at age 5, *p* = 0.04, at age 7, *p* < 0.01; MP vs. FT at age 3, *p* < 0.001, at age 5, *p* = 0.01, at age 7, *p* = 0.01, at age 11, *p* = 0.03) irrelevant (LP vs. FT at any age, *p* > 0.05)
Paz Levy D et al 2017 [56]	Israel	A population‐based cohort study	2	54,073 early term and 171,187 full term	5–18 years	Indication for labor induction, maternal characteristics (maternal pre‐pregnancy obesity), mode of delivery, neonatal low 5‐min Apgar score	Early term (37–38 weeks' gestation) vs. full term infants (≥ 39 weeks' gestation)	BMI percentile (overweight/obesity: > 85th)	Positive (overweight and obesity, early term = 104 (0.27%), full term = 261(0.20%), OR = 1.32(1.05,1.65), *p* = 0.02)
K.G Pringle et al 2019 [57]	Australia	Prospective cohort study	2	27 preterm and 200 term	1,2,3 years	Maternal characteristics, smoking, sex, maternal BMI, diet	Preterm infants (≤ 37 weeks' gestation) vs. term infants	BMI	Positive (BMI (kg/m^2^): preterm = 18.64 ± 2.24, term = 17.13 ± 1.65, *p* = 0.03)
Vasylyeva TL et al 2013 [58]	USA	Retrospective case control study	3	160 preterm	After 3 years	Demographic, maternal, family, neonatal, postnatal data (type of feeding)	Preterm infants (≤ 37 weeks' gestation, excluded overweight children)	BMI percentile	Positive (length of gestation (days): OR = 1.6 (1.1,2.4), *p* = 0.017)
Han J et al 2021 [59]	China	Cohort study	2	10,624 preterm	3, 6, 9, 12, 18, 24 months	Birth weight *z*‐ score, gender, intrauterine growth status	Preterm infants (<37 weeks' gestation)	BMI percentile (overweight: > 90th)	Positive (overweight risk at 3 months has 25.6%, at 24 months has 14.5%)
Forsum EK et al 2020 [60]	Sweden	Prospective cohort study	2	188 preterm (115 late preterm and 73 early preterm) and 253 term	4 years	Age (days) at measurement, birth‐related	Preterm infants (late preterm born after 224 days of gestation, early preterm born before 224 days of gestation) vs. term ( > 37 weeks' gestation)	BMI	Negative (BMI of the girls (kg/m^2^): full‐term = 15.6 ± 1.2, Early preterm = 14.8 ± 1.4, *p* < 0.05)
Kutar A et al 2023 [61]	India	Cross‐sectional study	2	170 preterm and 99 term	12–13 months	Birth anthropometry, nutritional status, sociodemographic details	Preterm infants (≤ 34 weeks' gestation) vs. term infants (≥ 37 weeks' gestation)	BMI *z*‐score	Negative (WHO *z*‐score mean of BMI for age: preterm = −0.2 ± 0.8, term = 0.5 ± 0.8, *p* < 0.0001)
Vinther JLet al 2023 [62]	Europe, North America, Australasia	Cohort study	2	253,810 infants	0–14 years	Gestational diabetes, gestational hypertension, maternal education, maternal height, maternal pre‐pregnancy BMI, maternal smoking during pregnancy, maternal age at child's birth, maternal ethnic background, preeclampsia, parity	Very preterm (28–33 weeks' gestation), late preterm (34–36 weeks' gestation), early term (37–38 weeks' gestation), full term (39–41 weeks' gestation), postterm (42–43 weeks' gestation)	BMI *z*‐score	Positive in preterm (BMI *z*‐score increase = 0.02SD(0.00,0.05), *p* < 0.05); irrelevant in adolescence (preterm BMI = term BMI (95% CI: −0.01,0.01), *p* = 0.9); positive in postterm (BMI *z*‐score increase = 0.02SD(0.00,0.05), 95% CI: 0.00; 0.05, *p* < 0.05)
Baran J et al 2022 [63]	Poland	Cohort study	2	123 preterm and 626 term	4–6, 7–11, 12–15 years	Maternal factors (mother's BMI), the role of the type of delivery	Preterm infants (<37 weeks' gestation) vs. term infants (≥ 37 weeks' gestation)	BMI percentile (overweight: 85th–95th; obesity: ≥ 95th)	Irrelevant (incidence of overweight and obesity at 4–6 years: preterm = 14.3%, term = 14.7%, *p* = 0.952; at 7–11 years: preterm = 12.5%, term = 13.8%, *p* = 0.799; at 12–15 years: preterm = 18.8%, term = 20.2%, *p* = 0.848)
Iguacel I et al 2018 [64]	Spain	Prospective cohort study	2	63 preterm and 968 term	6 years	Early life risk factors (maternal and paternal BMI, maternal smoking during pregnancy and maternal education), exclusive breastfeeding	Preterm (<37 weeks' gestation) vs. term (37–41 weeks' gestation)	BMI *z*‐score (overweight: > + 1SD; obesity: > + 2SD)	Irrelevant (underweight/normal vs. overweight/obese preterm: raw OR: 0.87 (0.37, 2.07); adjusted for confounding factors: 0.84 (0.35–2.01))
Parrott A et al 2022 [65]	USA	Prospective cohort study	2	112 preterm and 188 term	18, 24, 36, 42 months	Sociodemographic characteristics, infant feeding practices, household food security, caregiver BMI	Preterm (<37 weeks' gestation) vs. term (37–41 weeks' gestation)	BMI *z*‐score (possible overweight: 1SD to < 2SD; overweight and obesity: ≥ 2SD)	Irrelevant (WHO BMI‐for‐age *z*‐score category: overweight/obese: *p* = 0.23)

*Note:* BMI, Body Mass Index; EAR, Early adiposity rebound; FFM, Free fat mass; FMI, Fat mass index; FT, Full term; GA, Gestational age; LP, Late preterm; MD, Mean difference; MP, Moderately preterm; OR, Odds ratio; RR, Relative risk; SDS, Standard deviation scores; VP, Very preterm; WHO, World Health Organization; WHR, Waist–hip ratio; WHtR, Waist–height ratio.

Table 1 provides an overview of the general characteristics of the study population. Among the included studies, more than half (20 out of 28) focused on preterm children, employing various definitions, including: (1) preterm (< 37 weeks) (*n* = 10), (2) extremely preterm (< 32 weeks) (*n* = 1), (< =33 weeks) (*n* = 1), (3) late preterm (34–36 weeks) (*n* = 3), (4) moderately preterm (< 34 weeks) (*n* = 2) and (< 35 weeks) (*n* = 2) and (5) preterm across different gestational age categories (< 32, 32–33, 34–36 weeks) (*n* = 1).

The remaining studies (8 out of 28) examined children across a wider range of gestational ages, including two studies that encompassed the entire spectrum. Term was defined in these studies as 37–41 weeks (*n* = 1), and early term as 37–38 weeks (*n* = 1). Four studies specifically addressed post‐term births, defined as ≥ 42 weeks' gestation.

In all groups of preterm, full‐term newborns, and post‐term newborns, the results showed no significant differences in gestational age and obesity between men and women. However, some studies have found gender differences in specific situations. For example, at age 5, studies found that male preterm infants had lower body fat and fat‐free mass (FFM) than full‐term children [29], while another study reported that male preterm infants had lower BMIs than full‐term children [39].

For the definition of obesity, 17 articles used BMI and BMI percentile to define obesity, and defined obesity and overweight according to WHO and the standards of each country in the article; 8 articles defined the overweight and obesity interval according to BMI Z‐scores and the determined Z‐scores of children's age and gender. In addition, three articles incorporate FM and FFM to evaluate childhood obesity [43, 47, 48]. Some studies added data on weight, length or head circumference [47, 49], body fat [43, 50], EAR [51], WHR, WHtR [52] to compare the physical condition of children.

Most of the articles considered child factors and maternal factors. The relevant factors for most children include age, birth weight, gender, personal growth, race, region, and sex while the maternal factors mainly include maternal BMI, education level, maternal health status, and behavior during pregnancy, number of fetuses, and pregnancies. Some additional studies have taken into account infant feeding patterns [49, 51, 53], and paternal factors [39, 40, 42, 51, 54, 55] which are more commonly identified as covariates affecting infant obesity. An article considered socioeconomic factors [53], while others investigated physical activity status and adolescent status [48], induction of labor [56], family history [40]. These covariates need to be addressed and controlled for when studying child health and development to ensure the accuracy and reliability of research findings.

### 3.2 Preterm and Obesity

Among the 22 studies included in this review focusing on preterm‐born children [52, 57, 58, 59, 62], provided evidence supporting a positive association between preterm birth and overweight or obesity in children aged 3 months to 14 years. These findings are characterized by patterns of accelerated weight gain, rapid postpartum catch‐up growth, and changes in adipose tissue distribution among preterm infants. Specifically, one study [57] examined preterm infants and found that those born at a later gestational age had the highest risk of excess weight at 2 years old. Additionally, birth weight was consistently found to have a strong positive correlation with body weight in children as they grow older. In contrast, 12 studies [26, 27, 37, 38, 39, 40, 48, 52, 53, 55, 60, 61] reported lower BMI values among preterm‐born children in childhood (3 months–12 years old) compared to full‐term infants. Most studies reported that children born prematurely are thinner in the first 3 years of life than those born full‐term. One study [37] focused on preschool‐age children aged 5 to 8 years born prematurely and found significantly lower anthropometric characteristics. Additionally, another study noted varying impacts of gestational age on obesity outcomes, with preterm infants displaying lower average weights at 12 months but higher waist‐to‐hip and waist‐to‐height ratios at 14 years [52]. However, 6 other studies [47, 50, 51, 63, 64, 65] did not report statistically significant differences in BMI between preterm and full‐term children (age range from 3 months to 15 years). Specifically, one study [47] showed that late preterm infants had significantly higher average body weight and percentage fat mass at 1 month of age than full‐term infants, but no statistically significant differences between groups at 3 months of age.

### 3.3 Gestational Age at Full‐Term and Obesity

Among the studies focusing on full‐term children, 3 study [53, 56, 62] provided evidence supporting a positive association between gestational age in weeks and childhood overweight. The study [53] reported that very early‐preterm (< 33 gestational weeks) and somewhat early‐preterm (33–36 gestational weeks) infants had lower BMI compared to term (37–41 gestational weeks) infants. Another article [56] stated that overweight and obesity were more common in the early group (37–38 gestational weeks), and this finding was more pronounced in children older than 5 years. Additionally, one study [49] revealed a negative correlation between weight‐ and length‐growth rates and gestational age at 3 months of age.

### 3.4 Post‐Term and Obesity

Five studies [41, 42, 43, 53, 62] demonstrated a positive association between post‐term birth (≥ 42 weeks gestation) and overweight/obesity of children aged 9 months to 16 years, while one study [54] reported a negative association. Specifically, one study [41] found a positive association between post‐term birth and childhood overweight and obesity in male children, with post‐term boys being heavier than those born at term, particularly at 16 years of age. Likewise, a study investigating the metabolic effects of post‐term birth in childhood [43] revealed that post‐term‐born children aged 4 to 11 years had lower FFM and higher body fat, including increased central fat, trunk fat, and a greater proportion of androgynous to gynecological fat compared to controls. Another study [53] reported that post‐term (≥ 42 gestational weeks) infants had higher BMI compared to term infants. In contrast, a recent population‐based birth cohort study [54] found that post‐term pregnancy was associated with a higher risk of thinness, a lower risk of overweight/obesity, and lower growth indicators, including BMI‐for‐age Z‐score, weight‐for‐age Z‐score, and height‐for‐age Z‐score in preschool children aged 4 to 7 years. The other study [42] included 141,002 mother–child pairs, and found that post‐term birth was associated with elevated risks of obesity, overweight, and thinness in children aged 3–6 years, independent of sex.

## 4 Discussion

This scoping review aimed to explore the relationship between gestational age at birth and childhood obesity across the entire range. We identified 28 relevant studies published between January 1, 2000, and September 30, 2024. Overall, these studies showed substantial evidence that preterm birth can influence childhood obesity risk: 5 studies reported positive associations, 12 studies reported negative associations, and 6 studies found no statistical difference. However, Fewer articles focused specifically on term (*n* = 4) or post‐term (*n* = 6) births, yielding mixed and inconclusive findings in those groups.

This review revealed variations in the methods used by the studies included in the analysis. The majority of studies relied on BMI measurements to assess obesity, given its widespread use as an indicator of overall obesity in epidemiological studies and clinical practice. However, it is important to recognize that BMI has limitations in accurately distinguishing between lean and fat percentage, which affects its suitability for assessing adiposity [66, 67, 68]. Despite the estimation errors introduced by BMI, these errors are generally acceptable at the population level and account for a significant portion of the variation in children's body fat composition, ranging from 20% to 75% [66, 67, 69]. Furthermore, some studies incorporated additional measurements, such as plasma leptin concentration, which serves as an indicator of body fat, and fat levels as a marker of insulin sensitivity. Increased plasma leptin concentration has consistently been associated with obesity [60], while decreased plasma adiponectin concentrations have been observed [70]. These additional measurements offer complementary insights into the relationship between obesity and adiposity, providing a more comprehensive understanding of the relationship between gestational age and subsequent childhood obesity.

This review provides insights into the relationship between gestational age and obesity, revealing diverse findings across the included studies. Among the studies focusing on preterm children, the evidence was distinctly mixed: some reported a heightened obesity risk linked to accelerated catch‐up growth, while others suggested lower BMI in preterm‐born children early in life. Some studies on preterm‐born children have demonstrated positive associations with childhood obesity, characterized by accelerated weight gain, rapid postnatal catch‐up growth, and altered distribution of adipose tissue [71, 72, 73, 74]. While the exact mechanisms underlying the relationship between rapid weight gain and obesity in children remain unclear [74, 75, 76, 77, 78], several theories have been proposed to explain the increased risk of obesity in preterm‐born children. These include differences associated with preterm birth, such as perinatal programming [79], genetic factors [74], nutritional influences [74, 75, 76, 77] parental feeding patterns [78], and breastfeeding practices [78]. Notably, a study examining inflammatory proteins in preterm children [80] observed elevated concentrations of specific proteins associated with an increased risk of overweight and obesity. Among spontaneously delivered preterm infants, higher levels of VCAM‐1, an adhesion molecule, were associated with an increased risk of overweight, serving as a robust predictor for overweight and obesity in two instances. Moreover, β3‐adrenergic receptor polymorphism‐mediated lipolysis and insulin resistance resulting from hyperinsulinemia have been implicated in visceral fat proliferation [81] potentially contributing to weight gain and the development of infant and childhood obesity [74]. Rapid weight gain in infancy has also linked to a heightened risk of obesity in older children, and being overweight during nursery school has been found to increase the risk of obesity between the ages of 5 and 14 [82]. Another study conducted in Finland focusing on cardio‐metabolic risk factors among preterm‐born children [83] demonstrated higher percentages of body fat, waist circumference, and blood pressure in premature infants. Also, an American study showed that in a cohort of children born at extremely low gestational age, the risk of overweight and obesity is higher among those born to a mother with overweight or obesity, and among those who gained weight more rapidly during the first 2 years [84].

On the other hand, some studies on preterm‐born children have reported a negative association with obesity. This finding could be attributed to the limited energy and nutrient reserves in preterm newborns [85], weight loss observed in the initial months of life [86], challenges in the absorption of fatty acids due to the immaturity of their gastrointestinal tract function [87], and the duration of clinical intervention and intrauterine nutritional restrictions [86]. During the last trimester of pregnancy, the fetus primarily accumulates energy in the form of fat, glycogen, and nutrients. The growth pattern among preterm infants can vary in the initial months of life, and weight loss has been observed in these infants. This weight loss is inversely related to gestational age and directly associated with the duration of clinical intervention and intrauterine nutritional restrictions [86]. Additionally, premature newborns may face challenges in the absorption of fatty acids due to the immaturity of their gastrointestinal tract function [87]. These factors could all potentially contribute to the negative association observed between preterm birth and obesity in certain studies. The mixed results regarding the association between preterm birth and obesity may be attributed to variations in the age range of infants studied. Some studies focused solely on infants in the early months of life, which may explain why certain investigations reported a lack of significance. These studies found that preterm infants initially experienced a deficiency in growth nutrients during the first few days of life but subsequently exhibited early fat‐free mass accumulation within the first 3–4 months. Ultimately, they achieved similar fat mass values to those of full‐term infants [47, 53]. However, consistently across studies, it was observed that children and adolescents born preterm and aged at least 3 months exhibited a higher risk of OWO. For instance, in a study involving late preterm newborns [52], lower birth weights were reported compared to term births, but a higher body mass index (BMI) score was observed during adolescence. This association was mediated by infant weight gain at 12 months. These findings suggest that the pronounced risk of obesity in children may manifest more prominently at an older age, influenced by a combination of genetic factors, varied parental feeding practices, and exposure to obesogenic environments.

These diverse findings highlight the complexity of the relationship between preterm birth and subsequent obesity, evidenced by varied findings across studies. Generally, research indicates that while preterm children may initially have a lower risk of overweight and obesity due to factors like early nutritional deficits and growth challenges, this trend often reverses with age. By adolescence, many preterm individuals tend to exhibit increased BMI scores, potentially due to accelerated catch‐up growth, influenced by genetic factors, parental feeding practices, and their environments. These contrasting results highlight the complexity of determining how gestational age influences later obesity and underscore the need for additional large‐scale, longitudinal studies. This nuanced understanding also underscores the need for ongoing monitoring and tailored interventions that consider both the immediate and long‐term nutritional and health needs of preterm infants to mitigate their heightened risk of obesity as they age.

In addition to preterm‐born children, similar associations with obesity were observed for term and post‐term‐born children although fewer articles focused exclusively on these latter groups. Four studies specifically examined post‐term births (≥ 42 weeks), and two additional articles, which addressed a full range of gestational ages, also provided data on post‐term cohorts. Several studies, including cross‐sectional observational studies, population‐based cohort studies [49, 56, 88, 89], and the Keil Obesity Prevention study [90], have investigated the relationship between gestational age and childhood obesity in term‐born children. Consistently, these studies have found that children born early‐term (between 37 and 39 weeks) have a higher prevalence of overweight and obesity during childhood compared to completely full‐term (40 weeks) and late‐term (41 weeks) born children, and this increased incidence of overweight and obesity tends to persist into adolescence. Similar to preterm children, early‐term‐born children also exhibit postnatal catch‐up growth and rapid weight gain [56, 90, 91]. This phenomenon may be attributed to the accelerated growth experienced by infants with shorter gestation periods after birth compared to infants with longer intrauterine exposure [49].

The evidence on post‐term birth and obesity is limited with only 6 studies showing mixed results. Two studies demonstrated a clear association between higher BMI or obesity and greater gestational age, with the strongest effect observed in children born at 43 weeks of gestation or later [41, 43]. This suggests that post‐term‐born children experience accelerated postnatal catch‐up growth or rapid weight gain, similar to what has been reported in low birth weight or preterm children. More specifically, one study indicates an increased risk of obesity in post‐term boys but not girls [41]. However, contrasting findings have emerged from a recent large‐scale study conducted in China, focusing on preschool children [54]. This study found that post‐term pregnancies were associated with a lower risk of overweight/obesity, a higher risk of thinness, and overall lower growth parameters. These results suggest that post‐term pregnancies may hinder the long‐term development of children regardless of sex. Notably, although this study considered various potential confounding factors and adjusted for breastfeeding status and lifestyle factors during infancy and childhood, there is an important consideration to be noted. The prevalence of cesarean delivery was high in the sample, with 72.8% of term‐born and 63.1% of post‐term‐born children being delivered via cesarean section. This high prevalence of cesarean delivery raises the possibility that the findings of the study may be inconclusive. In another, more recent study with 141,002 Chinese preschoolers and a lower cesarean delivery rate (46.4% of term‐born and 45.3% of post‐term‐born), post‐term‐born preschoolers were found to have a higher risk of obesity, overweight, and thinness, independent of sex [42]. In summary, the evidence indicates a complex relationship between post‐term birth and obesity, with some studies showing a higher risk of overweight/obesity and poor lipid profiles among post‐term‐born children, particularly males, while others report a lower risk of overweight/obesity and hindered long‐term growth. Further research is needed to better understand these contrasting findings and elucidate the underlying mechanisms.

When considering gender differences, the overall findings suggest a consistent association between gestational age and obesity, with no significant variation between males and females across all groups of preterm, term, and post‐term‐born children. However, certain studies have identified gender disparities in specific contexts. For instance, at the age of 5 years, preterm male infants were found to have lower body fat and fat‐free mass (FFM) compared to full‐term children [27], while another study reported lower BMI in preterm males compared to their full‐term counterparts [39]. In contrast, in adulthood, males born preterm exhibited greater abdominal adiposity and higher BMI compared to males born full‐term [91]. The mode of delivery, particularly cesarean section for preterm birth, was associated with an increased percentage of overweight in boys [63]. Notably, a study investigating rapid weight gain in term‐born children found a positive correlation between weight gain and resting energy expenditure (REE) in boys [90]. Another study examining the prevalence of obesity across different ethnic groups reported that term‐born Pacific Island/Masoori children, predominantly male, had higher weights compared to term‐born Asian male children [91]. Gender differences in the association have also been observed in studies on post‐term‐born children. In Sweden, evidence showed a higher prevalence of overweight and obesity among post‐term‐born children, particularly males [41]. Another study revealed that both boys and girls born post‐term had an increased risk of obesity, with post‐term‐born males exhibiting a poor lipid profile characterized by higher ratios of total cholesterol to HDL cholesterol and total and LDL cholesterol levels [43].

When considering age differences and developmental trajectories, several studies have examined the association between gestational age and body mass index (BMI) at different ages. Some studies have found age differences, with preterm and early‐born babies having significantly lower BMIs at 9 months, while late‐born babies had higher BMIs compared to full‐term babies. Between the ages of 3 and 5, children born very early and early exhibited smaller declines in average BMI compared to term infants, while late‐born children maintained a higher trajectory. However, by the age of 5, the differences observed in very early and early‐born children at 9 months had diminished and were similar to term‐born children [53]. Another study showed that children born prematurely were shorter and weighed less, but by ages 5 to 7, they did not have increased fat mass or abdominal obesity [40]. A Chinese study demonstrated that the growth trajectory of premature infants in China consistently exceeded WHO standards from corrected age 40 weeks to corrected age 24 months [59]. The influence of age trajectory on body composition is a dynamic process that requires ongoing observation and intervention. One study [92] highlighted that premature infants tend to have a higher percentage of body fat compared to full‐term infants during the first 3 months. However, other studies have shown that the height and weight of preterm infants remain significantly lower than those born at term [93, 94, 95]. Similarly, another study [56] found that being overweight and obese was more common in the early‐term birth group (ages 0–18). This difference was particularly pronounced in children over 5 years of age, who exhibited higher rates of type I diabetes and obesity if born preterm. Furthermore, studies specifically investigating completely full‐term‐born children (ages 0–96 h) indicated that gestational age is associated with increased fat‐free mass and fat mass [88, 89].

It should be noted that, among the studies meeting our inclusion and exclusion criteria, most accounted for confounding factors such as birth weight and sex. While some studies also considered parental BMI or parental lifestyle factors, such as diet and activity, as covariates, they did not conduct further sub‐analyses specifically examining the impact of these factors. Given that parental BMI and family lifestyle choices can significantly influence childhood adiposity, the limited depth of analysis regarding these factors may restrict the generalizability of our findings and contribute to some of the inconsistencies observed. To address this gap, future research should not only include these variables more consistently but also explicitly analyze their influence. This approach will provide a more nuanced understanding of how gestational age interacts with parental and environmental factors to influence the trajectory of obesity risk from childhood into later life. Such detailed studies are crucial for developing targeted interventions that consider both genetic predispositions and modifiable lifestyle factors.

## 5 Strengths and Limitations

This scoping review offers unique insights into the relationship between gestational age at birth and childhood obesity. It is the first review to encompass gestational age across the full range, shedding light on the association between gestational age and the risk of obesity. The analysis includes high‐quality evidence that strongly supports the observed associations. However, it is important to acknowledge the limitations of this scoping review. Firstly, the inclusion criteria limited the review to studies published in English and from a number of databases, potentially introducing language and publication bias and omitting relevant data published in other languages or non‐publicly available sources. Moreover, focusing on studies published from 2000 onwards may have excluded valuable older research on the topic. Additionally, scoping reviews, unlike systematic reviews, do not typically involve a detailed assessment of study quality or risk of bias, which may impact the strength of the synthesized evidence. Scoping reviews prioritize breadth over depth, aiming to map the existing literature rather than provide an exhaustive analysis of individual studies. Despite these limitations, this scoping review has several strengths. It provides novel findings and includes high‐quality evidence. Moreover, it focuses specifically on the relationship between gestational age and obesity, contributing to a growing body of knowledge on the long‐term health effects of gestational age at birth. By identifying gaps in the literature and suggesting areas for further research, this review advances understanding in this field.

## 6 Conclusion

In conclusion, this scoping review provides valuable insights into the relationship between gestational age at birth and childhood obesity. The findings reveal a complex and diverse association, with most studies focusing on preterm birth and demonstrating either a positive or negative association with childhood obesity. However, evidence regarding term‐ and post‐term‐born children is limited. To gain a more comprehensive understanding of this relationship, future research should prioritize large birth cohort studies that cover the entire spectrum of gestational age, consider a wider range of confounders, and incorporate control groups for longitudinal evaluations of childhood obesity. Early identification of risk factors is crucial for effective prevention and management of the long‐term consequences associated with childhood obesity. This scoping review represents a significant advancement in understanding the potential link between preterm birth and childhood obesity, highlighting the importance of early interventions even before birth to improve outcomes and enhance the quality of life for affected children.

## Conflicts of Interest

The authors declare no conflicts of interest.

## Supporting information


**Appendix S1** Supporting information.
