# Supplementary material for: Association of Gestational Age in a Full Range With Childhood Overweight and Obesity: A Systematic Scoping Review
Source: Obes Rev. 2025 May 10;26(9):e13939. doi: 10.1111/obr.13939 (PMC12318906; doi:10.1111/obr.13939)
Supplement: Supplementary file 1 — Appendix S1 Supporting information. [file OBR-26-e13939-s001.pdf]

Association of gestational age in a full range with childhood overweight and obesity: a systematic scoping review

Yuantao Su BSc [1†] · Marini Ahmad Suhaimi BSc [2†] · Manish Prasad Gupta BSc[1] · Wenchong Du PhD[2*] · Jing Hua MD&PhD[1*]

[1] The Women's and Children's Health Care Department of Shanghai First Maternity and Infant Hospital, Tongji University School of Medicine, China

[2] NTU Psychology, School of Social Sciences, Nottingham Trent University, UK

† contributed equally

[*]Corresponding Author:

Wenchong Du PhD, Department of Psychology, Nottingham Trent University, Burton Street, Nottingham, UK, postcode: NG1 4BU (vivienne.du@ntu.ac.uk)

Jing Hua MD, PhD, Department of Mother and Children's Health Care, Shanghai First Maternity and Infant Hospital, School of Medicine, Tongji University, Shanghai 200092, China (Jinghua@tongji.edu.cn) and a visiting Professor at Nottingham Trent University

S1: Search Strategy for Systematic Scoping Review on Gestational Age and Childhood Overweight and Obesity

1. Google Scholar database searching terms – online (Google Scholar)

- Obesity
- Childhood
- Overweight
- Fatness
- Heavy weight
- Longitudinal growth
- BMI
- Adiposity
- Fat mass(FM)
- Free fat mass(FFM)
- 2+ Obesity
- 2+ Overweight
- 2+fatness
- 2+heavy weight
- 2+longitudinal growth
- 2+BMI
- 2+adiposity
- 2+fat mass (FM)
- 2+ free fat mass (FFM)
- Infant
- Infancy
- Fetus
- 1or 3 or 4 or 5 or 6 or 7 or 8 +20(21)
- 9/10+20(21)
- Perinatal/ perinatal risk factors
- Gestational age/ gestation at birth/ gestational weeks at birth
- Prematurity/preterm baby/preterm neonates/preterm born child
- Term/early term/full term/late term/+ child/neonates
- Post term + neonates/child/born child
- 11+25/26/27/28/29
- 12+25/26/27/28/29
- 13+25/26/27/28/29
- 14+25/26/27/28/29
- 15+25/26/27/28/29
- 16+25/26/27/28/29
- 17+25/26/27/28/29
- 18+25/26/27/28/29
- 19+25/26/27/28/29
- 23+25/26/27/28/29

2. PubMed searching terms

(Obesity[Title/Abstract] OR Overweight[Title/Abstract] OR Fatness[Title/Abstract] OR Heavy weight[Title/Abstract] OR Adiposity[Title/Abstract] OR Fat mass [Title/Abstract] OR Free fat mass [Title/Abstract])

AND (Childhood[Title/Abstract] OR Infant[Title/Abstract] OR Infancy[Title/Abstract] OR Fetus[Title/Abstract])

AND (Perinatal[Title/Abstract] OR Perinatal risk factors[Title/Abstract] OR Gestational age[Title/Abstract] OR Gestation at birth[Title/Abstract] OR Gestational weeks at birth[Title/Abstract] OR Prematurity[Title/Abstract] OR Preterm baby[Title/Abstract] OR Preterm neonates[Title/Abstract] OR Preterm born child[Title/Abstract] OR Term child[Title/Abstract] OR Term neonates[Title/Abstract] OR Early term child[Title/Abstract] OR Early term neonates[Title/Abstract] OR Full term child[Title/Abstract] OR Full term neonates[Title/Abstract] OR Late term child[Title/Abstract] OR Late term neonates[Title/Abstract] OR Post term neonates[Title/Abstract] OR Post term child[Title/Abstract] OR Post term born child[Title/Abstract])

3. Ovid Medline searching terms

(Obesity OR(Obesity OR Overweight OR Fatness OR Heavy weight OR Adiposity OR Fat mass OR Free fat mass).ab,ti,kw) and (Childhood OR Infant OR Infancy OR Fetus).ab,ti,kw and (Perinatal OR Perinatal risk factors OR Gestational age OR Gestation at birth OR Gestational weeks at birth OR Prematurity OR Preterm baby OR Preterm neonates OR Preterm born child OR Term child OR Term neonates OR Early term child OR Early term neonates OR Full term child OR Full term neonates OR Late term child OR Late term neonates OR Post term neonates OR Post term child OR Post term born child).ab,ti,kw

Limit to yr="2000 - 2024"
